# Open suprapubic versus retropubic prostatectomy in the treatment of benign prostatic hyperplasia during resident's learning curve: a randomized controlled trial

**DOI:** 10.1590/S1677-5538.IBJU.2014.0517

**Published:** 2016

**Authors:** Arie Carneiro, Paulo Sakuramoto, Marcelo Langer Wroclawski, Pedro Herminio Forseto, Alexandre Den Julio, Carlos Ricardo Doi Bautzer, Leonardo Monte Marques Lins, Andre Kataguiri, Fernanda Batistini Yamada, Gabriel Kushiyama Teixeira, Marcos Tobias-Machado, Antonio Carlos Lima Pompeo

**Affiliations:** 1 Hospital Israelita Albert Einstein, SP, Brasil; 2 Departamento de Urologia, Faculdade de Medicina do ABC, São Paulo, Brasil

**Keywords:** Prostatic Hyperplasia, Prostatectomy, Postoperative Complications, Prostate

## Abstract

**Purpose:**

This study compared the suprapubic (SP) versus retropubic (RP) prostatectomy for the treatment of large prostates and evaluated perioperative surgical morbidity and improvement of urinary symptoms.

**Materials and Methods:**

In this single centre, prospective, randomised study, 65 consecutive patients with LUTS and surgical indication with prostate volume greater than 75g underwent open prostatectomy to compare the RP (32 patients) versus SP (33 patients) technique. Results: The SP group exhibited a higher incidence of complications (p=0.002). Regarding voiding pattern analysis (IPSS and flowmetry), both were significantly effective compared to pre-treatment baseline. The RP group parameters were significantly better, with higher peak urinary flow (SP: 16.77 versus RP: 23.03mL/s, p=0.008) and a trend of lower IPSS score (SP: 6.67 versus RP 4.14, p=0.06). In a subgroup evaluation of patients with prostate volumes larger than 100g, blood loss was lower in those undergoing SP prostatectomy (p=0.003). Patients with prostates smaller than 100g in the SP group exhibited a higher incidence of low grade late complications (p=0.004).

**Conclusions:**

The SP technique was related to a higher incidence of minor complications in the late postoperative period. High volume prostates were associated with increased bleeding when the RP technique was utilized. The RP prostatectomy was associated with higher peak urinary flow and a trend of a lower IPSS Score.

## INTRODUCTION

Benign prostatic hyperplasia (BPH) is a condition that affects more than 50% of men over 60 years of age (1) and is the most common benign neoplasm in men. This pathology has gained even more notoriety due to the ageing population. A 7-fold increase in the elderly population is expected between the years 2000 and 2050 (2).

Clinical manifestations of the benign prostate growth include the onset of lower urinary symptoms that negatively impact quality of life in this population. An estimated 30% of men will require treatment for LUTS, and approximately 20% are likely to undergo surgical treatment (3, 4). Surgical treatment is reserved for refractory cases, as well as cases involving recurrent urinary retention, recurrent urinary tract infection, recurrent haematuria, bladder lithiasis, or upper urinary tract involvement (5).

There are several different surgical treatments for BPH, including transurethral resection of the prostate (TURP), open prostatectomy, laparoscopic and robotic prostatectomy, and other minimally invasive procedures (vaporization, transurethral incision, thermotherapy, ethanol ablation, holmium laser, among others). TURP is considered the gold standard surgical treatment for patients with prostate volumes less than 75g. For larger prostates (6) and in places with restricted access to newer technologies, open prostatectomy remains the standard procedure. Two open prostatectomy techniques have been described: the suprapubic (SP) and retropubic (RP) approaches are both widely performed all around the World (5). The advantages and disadvantages of these two techniques are commonly cited, but these reports have typically been based on case reports and personal experience of surgeons. To our knowledge, a randomised, prospective trial comparing the morbidity and functional outcomes between these two classic techniques has not been published.

## OBJECTIVE

This study sought to prospectively compare perioperative morbidity and improvement of symptoms of the RP and SP open prostatectomy techniques for treatment of BPH related LUTS during the resident's learning curve.

## MATERIALS AND METHODS

**Figure-1:**
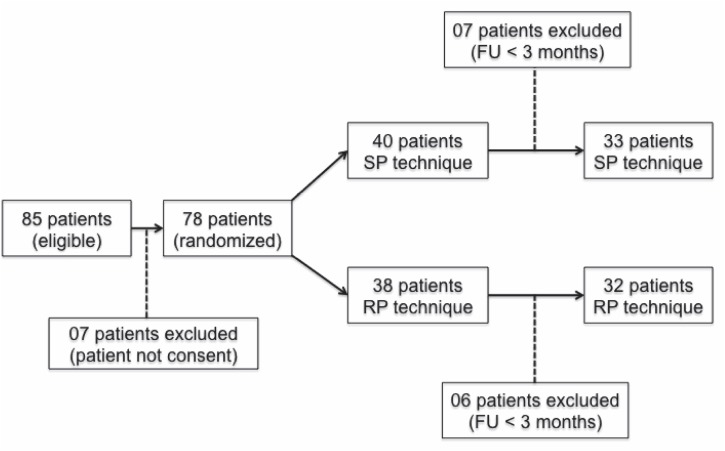
Study Flowchart.

We performed a single centre, prospective, randomized study comparing the two most widely performed techniques of open prostatectomy (RP and SP) as surgical treatment for patients with BPH related LUTS. We consecutively included 65 men with surgical indication for the treatment of BPH related LUTS and prostate volumes estimated by transabdominal ultrasound and digital rectal exam to be greater than 75grams (Figure-1). Patients with atonic bladder confirmed by urodynamic study and previous pelvic surgery were not eligible for the study. Patients with prostate cancer (elevated PSA or abnormal digital exam) suspicion had to have a negative prostate biopsy to be included. The study was approved by the local Ethics Committee and informed consent was obtained from each subject before inclusion in the study.

The initial assessment was performed using a protocol completed by the surgeon. The following parameters were included: demographic data, clinical examination, laboratory tests [PSA, hemoglobin (Hb)/hematocrit (Ht), urea and creatinine], imaging exams (abdominal USG), free flowmetry and International Prostate Symptoms Score (IPSS) questionnaire. The Charlson Index combined with age was used to quantify the burden of comorbidities (7). Follow-up visits were scheduled for 1, 3, 6 and 12 months after surgery. Symptom improvement (IPSS and free flowmetry) and complications were assessed at each visit.

The surgical technique was defined by simple randomization by sortition. All patients using anticoagulant therapy were informed to stop treatment 7 days before the procedure. The type of anaesthesia used was at the discretion of the anaesthesia team. The surgeries were performed by a first or second-year urology resident supervised by 3 senior surgeons (PS, MLW and PHFJ) with wide experience in both techniques. The techniques were previously standardised according to the Campbell-Walsh 10th edition (5). The indwelling catheter was scheduled to be removed on the seventh day after surgery in both techniques.

To access surgical morbidity, complications, blood loss estimation and necessity of transfusion were evaluated. Symptom improvement was evaluated via the IPSS score and free flowmetry. Blood loss estimation was calculated by the sum of the volume of blood present in the vacuum with the weight of the compresses, given a blood density of 1g/mL (8). The indication for blood transfusion followed the recommendations published by Roth et al. in 2010 (9). The Clavien-Dindo method was used to classify the complications (10) which were subdivided into intraoperative, immediate (during hospitalisation), early (before 1 month) and late (after 1 month) complications.

To achieve a p<0.05 and statistical power of 80%, we had to include at least 60 patients with 30 in each group. The student t-test, Mann-Whitney test, χ^2^ test, Pearson χ^2^ test, and multiple regression analysis were used for statistical analyses, performed with SPSS statistics software, Version 22 (IBM®).

## RESULTS

Sixty-five patients were included, 33 underwent surgery with the SP technique and 32 underwent surgery with the RP technique. The average age of the patients was 71 years. The main factors related to the indication for surgery were urinary retention (53.3%) and clinical treatment failure (21.5%). The mean follow-up was 12 months, and all patients attended at least the 3-month follow-up visit. Preoperatively, the groups were similar with respect to age, BMI, comorbidities (Charlson criteria), prostate size, IPSS and flowmetry (Table-1).

**Table 1:** Baseline and Demographic data.

	Total (n=65)	SP (n=33)	RP (n=32)	
Age - years (mean/SD)	71.13 / 8.3	72.06 / 8.63	70.13 / 8.09	*
BMI - kg/m^2^ (mean/SD)	25.60 / 4.08	26.08 / 3.64	25.08 / 4.51	*
Charlson Score (mean/SD)	3.56 / 1.10	3.53 / 1.08	3.61 / 1.12	*
Prostate Size-grams (mean/SD)	118.68 / 52.13	116.82 / 56.93	120.5 / 47.94	*
IPSS (pre-op)(mean/SD)	25.08 / 14.01	26.57 / 18.28	23.33/6.29	*
Indwelling urinary catheter (%)	69.50%	69%	70%	*
Hb – mg/dL (mean/SD)	13.98 / 1.33	13.78 / 1.35	14.16 / 1.31	*
Ht – % (mean/SD)	42.28 / 3.87	41.6 / 4.4	42.9 / 3.27	*
Flowmetry – mL/s (mean/SD)	4.3 / 17	4.31 / 26.54	7.9 / 20.34	*
Follow-up - months (mean/SD)	12.33 / 9.34	13.95 / 8.99	10.44 / 9.64	*

* p>0.05

Eleven cases involved bladder calculus (SP: 6 versus RP: 5), all were managed in the same surgical procedure, without any additional intercurrence. The mean operating time was 126.5 minutes (SP: 125.65 versus RP: 127.41 minutes; p=0.75), and was not statistically different between the two techniques. Bladder irrigation (SP: 2.74 versus RP: 2.74 days, p=1), and abdominal drainage (SP: 2.96 versus RP: 3.32 days; p=0.64) were also similar between the groups. The indwelling bladder catheter placement time was shorter in the group that underwent RP prostatectomy, however, the difference was not statistically significant (SP: 10 versus RP: 7.92 days; p=0.12) (Table-2).

**Table 2:** Peri and postoperative assessment and early complications.

		Total (n=65)	SP (n=33)	RP (n=32) 127.41 / 31.95	p
Time of surgery- minutes (mean/SD)		126.50 / 36.07	125.65 / 40.06	127.41 / 31.95	*
Hospital stay- days (mean/SD)		4.60 / 2.19	4.52 / 1.37	4.67 / 2.76	*
Irrigation - days (mean/SD)		2.74 / 1.54	2.74 / 2.16	2.74 / 1.51	*	
Drain - days (mean/SD)		3.15 / 1.32	2.96 / 1.02	3.32 / 1.54	*
IPSS Post-op (mean/SD)		5.56 / 5.43	6.67 / 6.86	4.14 / 2.25	0.06
**Flowmetry post-op**					
	Maximal flow - mL/seg (mean/SD)	19.78 / 11.23	16.77 / 11.24	23.03 / 10.73	0.008
	Final volume - mL (mean/SD)	192.00 / 100.06	181.15 / 64.05	203.77 / 130.70	*
**Blood loss**					
	Hb (48 hours) – mg/dL (mean/SD)	9.93 / 1.47	10.49 / 1.47	9.93 / 1.47	*
	Ht (48 hours) - % (mean/SD)	29.95 / 4.3	31.44 / 4.7	29.95 / 4.3	*
	Estimative of Bleeding- mL (mean/SD)	1044.25 / 619.74	927.51 / 554.03	1156.82 / 667.68	*
**Transfusion**					
	Yes	3.90%		8.30%	
	No	96.10%	100.00%	91.70%	*

* p>0.05

In the evaluation of symptoms, both techniques demonstrated a significant decrease in IPSS (SP: 26.57 to 6.7, p<0.001 and RP: 23.33 to 4.14, p=0.001) and a significant increase in urinary free flow (SP: 4.3 to 16.77mL/s, p=0.02 and RP: 7.9 to 23.03mL/s, p<0.001), when comparing the pre-operative visit to the last follow-up visit. The IPSS after treatment was similar in both groups, however the RP group had a trend of lower IPSS scores (SP: 6.7 versus RP: 4.14; p=0.06) and higher peak urinary flow (SP: 16.77 versus RP: 23.03mL/s, p=0.008) (Table-2).

The haematological parameters were similar between the groups before the procedure (Table-1). The peri-operative blood loss estimation was similar between the groups, with an average intraoperative blood loss of 1044mL (SP: 927.5 versus RP: 1156mL, p=0.14) and an Hb average after 48 hours of 9.93mg/dL with both techniques showing a significant decrease from pre-operative (SP: 13.78 to 10.49mg/dL, p<0.001 and RP: 14.16 to 9.9mg/dL, p<0.001), however both groups presented a similar Hb after 48 hours (SP: 10.49 versus RP: 9.9mg/dL, p=0.11). Blood transfusion during surgery was required for three patients treated by SP and one individual treated by the RP technique (Table-2).

Forty-nine complications were identified, with 4 intraoperative, 12 immediate, 11 early and 22 late complications. Seventeen complications were considered severe (Clavien 3 or 4), and 32 were considered minor (Clavien 1 or 2) (Table-3). Complications were more prevalent in patients undergoing the SP technique (SP: 32 versus RP: 17, p=0.002). The number of late complications was also higher in the SP group (SP: 17 versus RP: 5, p=0.004), for late complications, 7 were classified as severe (Clavien 3) in the SP group, and 1 was classified as severe in the RP group.

**Table 3:** Complications according to Clavien-Dindo classification.

		All	SP	RP
**Intraoperatively**				
	Bleeding (II)	3(0.06)	3	0
	Severe Bleeding (IV)	1(0.02)	0	1
**Immediate**				
	ARF (I)	1(0.015)	0	1
	Delirium (I)	2(0.03)	0	2
	Persistent Bleeding (II)	1(0.015)	0	1
	Wound Infection (II)	2(0.03)	0	2
	Recurrent hematuria (IIIa)	1(0.015)	1	0
	Retained Clots (IIIB)	5(0.07)	2	3
**Early**				
	Urinary Incontinence (ID)	3(0.04)	2	1
	UTI (II)	5(0.07)	4	1
	AUR (IIIA)	3(0.04)	3	0
**Late** *				
	Hydrocele(I)	3(0.04)	2	1
	Urinary Incontinence (ID)	1(0.015)	0	1
	Hematuria (I)	1(0.015)	1	0
	Overactive Bladder (II)	2(0.03)	2	0
	UTI (II)	6(0.09)	4	2
	Wound Infection (II)	1(0.015)	1	0
	AUR (IIIA)	3(0.04)	3	0
	Urethral stricture (IIIB)	2(0.03)	2	0
	Bladder Neck Stricture (IIIB)	2(0.03)	1	1
	Vesico-cutaneus fistula (IIIA)	1(0.015)	1	0

* p=0.04

**UTI =** Urinary Tract Infection; **ARF =** Acute Renal Failure; **AUR =** Acute Urinary retention

In the analysis of the severity of complications, two cases should be highlighted for their severity. The first case involved a patient who underwent SP prostatectomy and developed a vesico-cutaneous fistula requiring prolonged vesical indwelling catheterisation. The second case underwent RP prostatectomy and developed significant intraoperative bleeding due to inadvertent injury of the retropubic vessel, with hypovolemic shock and the need for a transfusion of 4 units of packed red blood cells.

In the subgroup of patients with prostate volumes estimated to be greater than 100grams, those in the RP group exhibited significantly more bleeding (mean estimated blood loss, SP: 863mL (SD: 462) versus RP: 1313mL (SD: 671), p=0.003) compared to SP group. The incidence and severity of complications were similar between the techniques.

In patients with prostate volumes estimated to be between 75 and 100g, the estimated peri-operative bleeding was similar between the groups. However, the SP group exhibited a higher incidence of late complications (SP: 17 versus RP: 5, p=0.004).

We observed that 15.4% of our patients required some re-endoscopic surgical intervention, 50% of these for evacuation of clots.

## DISCUSSION

Open prostatectomy is the most effective and durable method for controlling symptoms associated with BPH and is widely performed, mainly for patients with bulky prostates and in countries where access to technology is limited. Thus, understanding the details of this procedure is of great importance.

The risk of bleeding and the complications of the procedure vary significantly according to the centre performing the surgery. Currently, due to the expansion of endourological methods, open surgery has been less studied. Thus, there is a lack of studies with adequate methodology and standardised reporting of complications as well as analyses of the learning curve.

In our study, we prospectively evaluated a series of patients that underwent open prostatectomy during the residents learning curve comparing the surgical morbidity and functional outcomes between the two most widely used techniques. To our knowledge, there has been no study in the World literature performing such a comparison. Both techniques used in this study are well established and are still performed even in places where TURP is widely used for patients with massive prostates (11).

The RP technique was described by Terrance Millin and published in The Lancet in 1945. According to Campbell's Urology 10th edition, this technique has the advantages of better control of bleeding, better visualisation of remnant adenoma, no effect on the bladder, and good anatomic exposure of the prostate, however, the technique does not allow for direct access to the bladder, an important factor in cases with lithiasis and bladder diverticula (5). The SP technique (transvesical) was described by Eugene Fuller in 1894 and popularised by Peter Freyer in 1900-1912. According to Campbell's Urology 10th edition, this technique has the advantage of being able to directly visualise the bladder and bladder neck but has a vision deficit of the prostatic apex and difficulty in controlling bleeding (5).

Conversely, in our study, the RP technique was significantly related to intraoperative bleeding when the prostate volume was greater than 100g. On the other hand, there was no difficulty in treating bladder stones through this approach. The presence of bladder lithiasis does not present a limiting factor for the RP technique, in our series, 5 patients with bladder lithiasis were treated with the RP technique without increased morbidity.

In the literature, we found several cases of SP prostatectomy and RP prostatectomy and a few studies that compared open prostatectomy with fewer invasive procedures. Ou et al. published a randomized series of 80 cases comparing SP prostatectomy with TURP, suggesting that the improvement in quality of life and the IPSS of the patients undergoing open prostatectomy were significantly better than of patients who underwent TURP (12).

In this study, both techniques were effective in improving the symptoms of the lower urinary tract, nevertheless the RP technique was related with statistically significant higher peak urinary flow (SP: 16.66 versus RP: 23.03mL/s, p<0.05) and a trend of lower IPSS (SP: 6.67 versus RP 4.14, p=0.06), although these findings may not represent a clinical difference. However, there are no scientific data to support this finding, one may hypothesize that bladder incision in the SP technique may have impact in the bladder function and therefore be related with the above findings.

A report of a modified Millin technique which compares this approach to the standard SP technique has been previously published by Dall’Oglio et al. (13). The authors concluded that the modified technique can significantly control bleeding during and after surgery, and reduce transfusion rates, when compared to the classic transvesical prostatectomy. However, a modified technique was performed in that study and the global morbidity and effectiveness were not addressed.

In our study, the blood loss estimation in the total population was 1044mL and was similar between the techniques. Although considered high, when compared to series within the World literature, there was no significant drop in the haematocrit and little increase in the transfusion rate, consistent with approximately 5 to 8% of cases reported in the literature (14). Our increased blood loss may be explained by the fact that residents at the beginning of their learning curve performed the procedures.

Although bleeding was similar between the techniques, the transfusion requirements were higher in the SP group, however, in the RP group, the required number of red blood units in the event of transfusion was significantly higher (four units for the RP versus 1 unit in each SP case), indicating greater difficulty in controlling bleeding that occurred in the RP technique.

The analysis of the subgroup of patients with massive prostates (>100g) revealed increased bleeding in the RP group (RP: 1313mL versus SP: 863mL, p=0.04). This increase may also be explained by the technical difficulty in controlling the lateral pedicle in larger prostates.

Moslemi et al. reported that open prostatectomies offer the advantage of low rates of re-treatment when compared to TURP (15), although might be related with more re-interventions. In our study, this pattern could be also found, since 15.4% of our patients underwent re-endoscopic surgical intervention. This may be partially explained by the lack of a specialized multi-professional team, that often inadvertently stops bladder irrigation.

Our study demonstrated that both techniques have a high complication rate (75% of patients had some type of complication, whether intraoperative, immediate, early or late). However, complications were typically easy to handle (Clavien 1 or 2), and there was only one case of Clavien 4 complication. In the past, given the lack of standardisation of complications, these events would likely not have been counted as complications of TURP and prostatectomy series, and can explain the lower incidence reported in previous publications (16, 17).

The SP technique was related to a higher number of complications (p=0.002). In the analysis of late complications, the incidence was significantly higher in the subgroup undergoing the SP technique (p=0.004), and severe complications (Clavien 3 or 4) were more frequent in the SP group (SP: 4 versus RP: 1). The incidence and severity of immediate and early complications were similar between the two groups. We suspect that the increase in the number of late complications in the SP technique was related to the bladder incision, which does not occur in the RP technique. In patients with bulky prostates, the incidence and severity of complications were similar between the groups.

In developed countries, newer therapies such as laser enucleation, laparoscopic and robotic surgery have been applied with good results, improving the estimated blood loss, surgical morbidity and hospital length of stay. However, these technologies are expensive and require a long learning curve that may limit their feasibility worldwide (18, 19).

Open prostatectomy, while a more aggressive management modality to treat BPH, is still widely performed, mainly in places with limited access to technology. One aspect of this study is that all surgeries were performed by residents, which may explain the blood loss and the rate of complications greater than expected, even though supervised by a senior surgeon with great experience in both techniques. Another limitation of the study was the absence of data regarding the amount of resected prostatic tissue, although the whole adenoma was supposedly resected during both procedures. Although the statistical power of 80% was reached, with a population of more than 60 patients, a larger number would be warranted to consolidate our findings.

## CONCLUSIONS

RP and SP techniques are safe and effective in the surgical treatment of BPH related LUTS, even during the learning curve of residents. The choice of technique should be based on the experience and training of each surgeon. The RP technique was associated with higher statistical improvement of urinary symptoms, however, it probably does not represent clinical significance. Although the incidence of complications was high, they were typically easy to handle (Clavien 1 or 2). The SP technique was associated with a higher incidence of late complications, and the RP technique increased the risk of excessive bleeding in massive prostates (greater than 100grams). A similar study should be conducted in other centres to confirm these results.
